# The Hidden Power of the Secretome: Therapeutic Potential on Wound Healing and Cell-Free Regenerative Medicine—A Systematic Review

**DOI:** 10.3390/ijms26051926

**Published:** 2025-02-23

**Authors:** Jhon W. Prado-Yupanqui, Lourdes Ramírez-Orrego, Denny Cortez, Victor Juan Vera-Ponce, Stella M. Chenet, Juan R. Tejedo, Rafael Tapia-Limonchi

**Affiliations:** 1 Instituto de Investigación de Enfermedades Tropicales, Universidad Nacional Toribio Rodríguez de Mendoza de Amazonas, Chachapoyas 01001, Peru; jhon.prado@untrm.edu.pe (J.W.P.-Y.); lourdes.ramirez@untrm.edu.pe (L.R.-O.); denny.cortez.epg@untrm.edu.pe (D.C.); victor.vera@untrm.edu.pe (V.J.V.-P.); stella.chenet@untrm.edu.pe (S.M.C.); jrtejhua@upo.es (J.R.T.); 2 Facultad de Medicina, Universidad Nacional Toribio Rodríguez de Mendoza de Amazonas, Chachapoyas 01001, Peru; 3 Departamento de Biología Molecular e Ingeniería Bioquímica, Universidad Pablo de Olavide (UPO), 41013 Seville, Spain; 4 Biomedical Research Network for Diabetes and Related Metabolic Diseases (CIBERDEM), Instituto de Salud Carlos III, 28029 Madrid, Spain

**Keywords:** MSC, regenerative medicine, secretome, wound healing

## Abstract

Various types of wounds represent a persistent healthcare burden that demands innovative and effective therapeutic solutions. Innovative approaches have emerged that focus on skin regeneration with minimal side effects. One such method is cell-free therapy that utilizes the secretome of human mesenchymal stem cells (hMSCs) as a promising alternative to traditional cell-based therapies, leveraging a complex mixture of bioactive molecules, including growth factors, cytokines, and extracellular vesicles, to accelerate tissue regeneration. This systematic review synthesizes the findings of 35 studies evaluating the impact of hMSC-derived secretomes on wound healing, with a focus on their regenerative, immunomodulatory, and angiogenic effects. The influence of MSC sources (adipose tissue, bone marrow, umbilical cord) and culture conditions on secretome composition and efficacy in the cutaneous wound healing process is examined, highlighting their therapeutic potential in regenerative medicine. This review also explores emerging preclinical and clinical applications, highlighting promising results, such as enhanced fibroblast proliferation, reduced inflammation, and improved extracellular matrix remodeling. In addition, advances in secretome-based biomaterials, including hydrogels and scaffolds, which optimize therapeutic delivery and efficacy are discussed. Despite the growing body of evidence supporting the safety and efficacy of secretomes, challenges remain regarding standardization, large-scale production, and clinical validation. This review highlights the potential of MSC-derived secretomes as a next-generation cell-free approach for wound healing and regenerative medicine.

## 1. Introduction

In the context of wound healing, the MSC secretome has shown promising results by influencing the four main phases of the process: hemostasis, inflammation, proliferation, and remodeling [1]. This effect is achieved through the action of key anti-inflammatory cytokines and growth factors, such as VEGF and FGF, as well as miRNAs carried in microvesicles, which promote cell proliferation, angiogenesis, and extracellular matrix reorganization [2]. On their own, MSC-derived exosomes have demonstrated their ability to stimulate the proliferation and migration of fibroblasts and keratinocytes, efficiently supporting tissue regeneration and minimizing scar formation [3].

The mesenchymal stem cell (MSC) secretome is a complex mixture of bioactive factors and extracellular vesicles (EVs) that play essential roles in cellular communication and tissue regeneration [4]. This secretome can be obtained from various sources, such as the umbilical cord (UC-MSC), bone marrow (BM-MSC), and adipose tissue (AD-MSC), among others, broadening its therapeutic potential in regenerative medicine [5]. Its composition includes cytokines, chemokines, growth factors, immunomodulatory molecules, and extracellular vesicles, such as exosomes and microvesicles, making it a key mediator in various biological processes [5,6].

The biogenesis of the secretome occurs through classical and non-classical intracellular mechanisms, including protein translocation, exocytosis, and vesicle or exosome encapsulation [6,7,8]. In classical mechanisms, newly synthesized proteins are transported to the lumen of the endoplasmic reticulum (ER), where they undergo post-translational modifications, such as signal peptide cleavage, folding, and glycosylation. This process is guided by an N-terminal signal sequence, which directs ribosomes to the ER during polypeptide synthesis [8]. Subsequently, the modified proteins are transferred to the Golgi complex for final processing and released into the extracellular environment via exocytosis [9,10].

In non-classical secretion mechanisms, certain molecules, such as growth factors and cytokines, which play a key role in immune response, angiogenesis, cell growth, and differentiation, can be released without the involvement of the ER–Golgi complex [8]. This release can occur directly through the cell membrane or encapsulation in extracellular vesicles (EVs) [10]. EVs, which are part of the secretome, contain lipids, messenger RNA (mRNA), microRNA (miRNA), and bioactive proteins, significantly contributing to the regulation of cellular signaling pathways and tissue repair [7,11,12,13]. Their classification is based on their origin and size [14].

**Figure 1:**
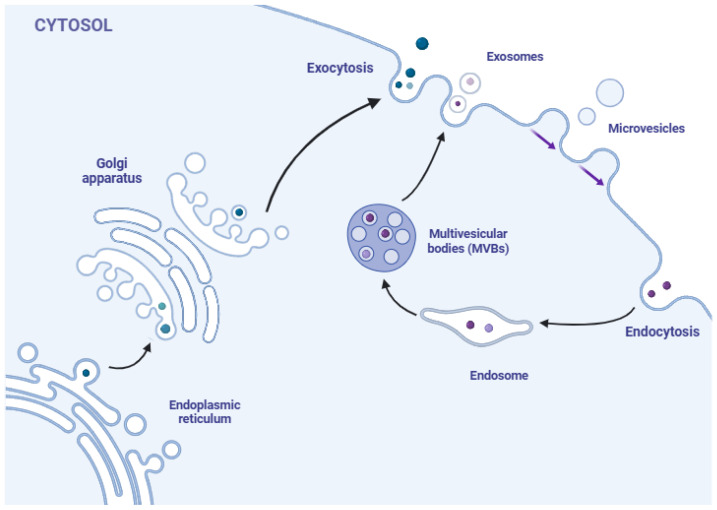
Biogenesis of secretome.

Microvesicles, which range from 100 to 1000 nm in diameter, constitute the largest fraction of EVs and transport their content to nearby or distant cells through interactions with microtubules and SNARE proteins. They originate through membrane budding or protrusion [15,16,17,18]. In contrast, exosomes measuring between 30 and 100 nm are surrounded by a lipid bilayer and released through exocytosis [19]. They are generated within the cell when endosomes fuse with endocytic vesicles, forming multivesicular bodies (MVBs), which, upon fusion with the plasma membrane, release their content into the extracellular environment [15,17]. EVs can be taken up by recipient cells through ligand–receptor interactions, facilitating their internalization and subsequent release of their contents into the cytoplasm, thereby modulating cellular activity. Additionally, EVs have a prolonged circulating half-life (Figure 1) [9,15,16].

The secretome of mesenchymal stem cells (MSCs) represents an innovative alternative to cell transplantation, as its therapeutic action is based on the release of bioactive factors rather than the integration of cells into the recipient tissue [20]. This strategy circumvents the limitations associated with the storage and handling of viable MSCs, reducing costs and the need for specialized infrastructure [21]. Moreover, the secretome minimizes the risk of immune rejection and the formation of undesired cell masses, thereby enhancing treatment safety [20,21]. Recent studies have shown that biomaterials functionalized with secretome can improve arteriovenous fistula maturation, decrease neointimal hyperplasia, and modulate inflammatory responses, promoting tissue regeneration [20]. In particular, the use of bioabsorbable polymeric scaffolds loaded with MSC secretome has demonstrated positive effects in preclinical models of chronic kidney disease, highlighting its potential in advanced wound healing therapies and regenerative medicine [21].

The secretome can also be optimized through cellular preconditioning, such as exposure to hypoxia or inflammatory stimuli, enhancing its bioactive content [22]. For these reasons, the secretome emerges as a promising tool in regenerative therapies, particularly in wound healing, with significant potential for clinical application [13]. This systematic review aims to evaluate the impact of the mesenchymal stem cell secretome on the wound healing process, highlighting its therapeutic potential in regenerative medicine.

## 2. Material and Methods

This systematic review was performed according to the Preferred Reporting Items for Systematic Reviews and Meta-analyses (PRISMA-2020) statement [23]. Furthermore, it was recorded on the Open Science Framework (OSF) (Viginia, USA) registry, with access code https://osf.io/4yqhc. The literature search was carried out in Scopus, Web of Science, PubMed, and EMBASE databases using the terms “mesenchymal stem cells”, “wound healing”, and “secretome”. The selected query identified original articles reporting preclinical and clinical applications focused on skin wounds treated with factors secreted by MSCs as intervention. The search was limited to articles published in English. In addition, the electronic search was complemented with a manual review of the reference lists.

- Inclusion criteria

**Figure 2:**
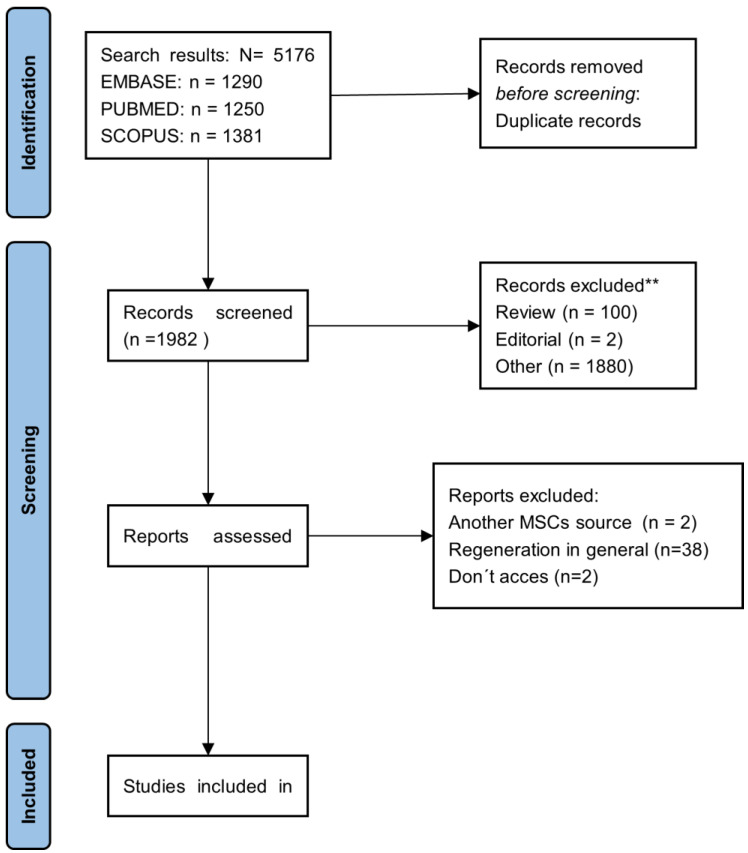
The PRISMA-2020 flowchart of the study selection process for the systematic review.

This study was conducted with the following inclusion criteria: original research; preclinical and clinical studies; secretome derived from adipose tissue, bone marrow, umbilical cord, umbilical cord blood; and studies that evaluated the impact of secretome on cutaneous wound healing. As exclusion criteria, reviews and systematic reviews, non-original studies, editorials, and book chapters were excluded. Studies focused exclusively on extracellular vesicles and secretomes of hMSC isolated from other human sources (e.g., placenta and menstrual blood, among others) and those of animal origin were also excluded. Figure 2 represents the PRISMA flowchart for the systematic review.

- Data Collection

Three independent reviewers (J.W.P.-Y., L.R.-O., and D.C.) assessed full-text articles previously screened in RAYYAN (Doha, Qatar). The extracted data included the title, objective and/or hypothesis, methodology and study design, outcomes, source of mesenchymal stem cells (MSC), year of publication, and country of origin. Studies with unclear or missing results were excluded from the review. The authors of articles without full access were contacted to request complete information regarding their research. If no response was received, those articles were also excluded. The three reviewers (J.W.P.-Y., L.R.-O., and D.C.) made the decisions regarding inclusion and exclusion collaboratively.

- The worldwide commercial situation for MSC secretome

In addition to conducting a systematic review, we evaluated the commercial landscape of MSC secretome-based products that have entered the cell therapy market. We gathered publicly available information on MSC secretome-based products available for sale worldwide. Some of these products include interventions utilizing stem cell secretome in hospitals and off-the-shelf secretome products supplied by third-party companies. Our search query terms included “wound healing”, “secretome”, and “mesenchymal stem cells”, and we utilized Google Search and Google Maps for this purpose. The search was performed between November and December 2024.

## 3. Results

**Figure 3:**
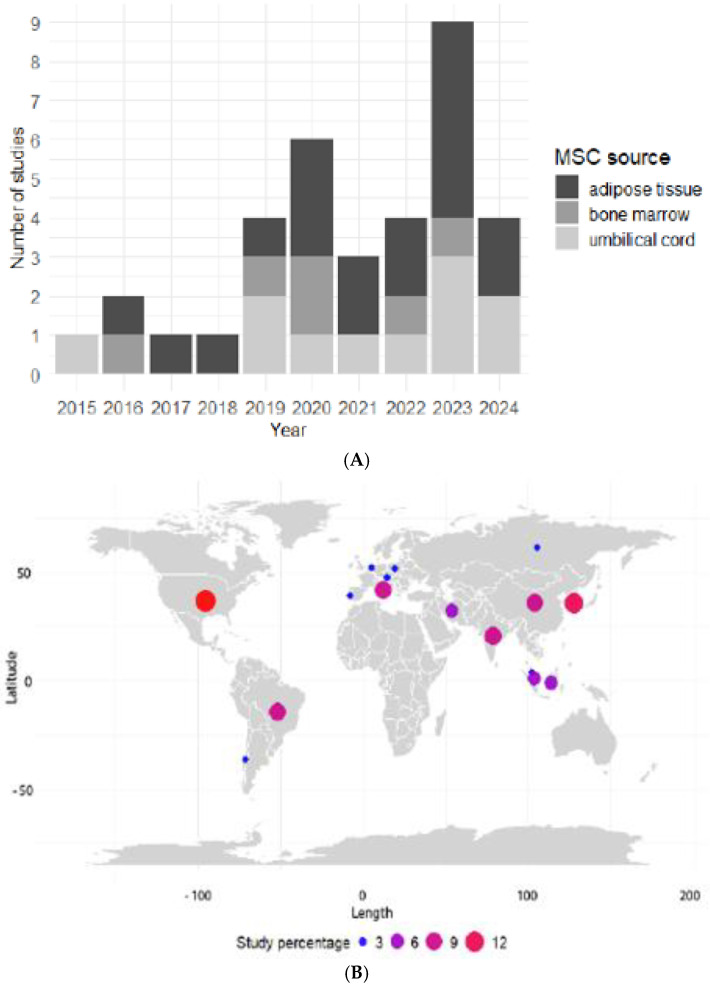
Main features of the selected articles. Trend of secretome studies from different mesenchymal stem cells (MSC) sources (**A**), and geographical distribution of research on using MSCs secretome in wound healing (**B**).

A total of 35 articles fulfilled the eligibility criteria. Over the past ten years, the number of reports on the secretome of human mesenchymal stem cells (hMSCs) has been increasing, with a peak in 2023. The studies predominantly focused on three major MSC sources: adipose tissue, bone marrow, and umbilical cord. There are significantly more studies on the secretomes derived from adipose tissue MSCs than other sources, followed by umbilical cord MSC secretomes. In contrast, few studies utilized secretomes from bone marrow MSCs (Figure 3A). From a manufacturing perspective, adipose tissue and umbilical cord sources are preferred due to their higher yield and other quality attributes that are important for various applications. In terms of geographic distribution, Asian countries lead with eighteen studies (51%), followed by European countries with eight studies (23%), the USA with five studies (14%), and South America, represented by four studies conducted in Brazil (11%) and one in Chile (2%) (Figure 3B).

### 3.1. Source of MSCs

The secretome derived from the MSCs plays an essential role in their therapeutic effects, as it contains a complex mixture of proteins, growth factors, cytokines, and extracellular vesicles that promote tissue repair, modulate immune response, and support tissue regeneration [24,25,26]. Human umbilical cord-derived mesenchymal stem cells (hUC-MSCs) are obtained from the umbilical cord, which makes their collection minimally invasive and ethically acceptable [24]. The secretome of hUC-MSCs contains a variety of bioactive factors, including growth factors such as VEGF (vascular endothelial growth factor), FGF (fibroblast growth factor) [27], insulin-like growth factor (IGF), transforming growth factor-beta (TGF-β) [28], and hepatocyte growth factor (hHGF) [25], which are crucial for angiogenesis and tissue repair, as well as modulating the immune response and promoting cell survival and proliferation [27,29,30]. This systematic review identifies the secretome, or conditioned medium, of MSCs derived from the human umbilical cord, with a specific emphasis on Wharton’s jelly as a source in some cases [24,27,28,29,31,32,33,34,35]. Studies show that the secretome of human hUC-MSCs has a positive effect on the healing of chronic ulcers, including diabetic and leprosy-related ulcers [29], emphasizing its potential for therapeutic use [35].

Secretomes derived from adipose tissue MSCs are recognized for their regenerative properties. Some reports have demonstrated positive effects on granulation tissue formation and vascularization, which helps prevent fibrosis in pressure ulcers [26]. By characterizing these secretomes, key trophic factors have been identified that enhance the regeneration process [36,37]. Additionally, advancements in production methods have enabled the development of enriched secretomes that promote healing without leaving visible scars [38]. Furthermore, the incorporation of these secretomes into innovative hydrogels and dressings has improved their effectiveness in treating chronic wounds and dermonecrosis models [39,40]. The secretome of bone marrow-derived MSCs has also been explored as an effective source for treating difficult wounds. These cells have demonstrated the ability to restore cellular autophagy in diabetic wounds through specific molecular pathways, such as HIF-1α/TGF-β1/SMAD [41]. In combination with technologies such as enriched hydrogels and low-level laser therapies, their secretome has demonstrated significant acceleration in wound healing [42,43]. Furthermore, their ability to reverse adverse conditions such as hypoxia and low sleep availability, common factors in chronic wounds, has been shown [44].

### 3.2. Methods for Obtaining Secretome

Mesenchymal stem cell (MSC) secretome is obtained through specific methods depending on the cell source, with variations in culture medium, incubation conditions, and final processing. In general, MSCs are cultured until reaching 70–90% confluence, at which point the standard medium is replaced by a sleep-free medium to avoid interference from exogenous proteins [26,41]. The incubation time varies significantly, from 24 h in studies with bone marrow cells (BM-MSC) [36] to 7 days in adipose tissue models (AD-MSC) [30]. For secretome processing, centrifugation techniques at 1500–2000× *g* are used to remove cellular debris [45], followed by ultrafiltration with a threshold of 3–5 kDa to concentrate the soluble bioactive factors [29,46]. In some studies, lyophilization is applied to preserve biological stability and facilitate storage [25]. Furthermore, in hypoxia models (5% O_2_), the secretome of BM-MSC and UC-MSC showed a higher concentration of angiogenic factors, such as VEGF and bFGF, promoting healing [27,39]. In the case of the secretome of AD-MSC, the presence of exosomes has been emphasized by isolation with the exoEasy Maxi kit (Qiagen, Hong Kong, China) and their characterization with NanoSight LM10 [47]. On the other hand, in studies with UC-MSC, the use of media without phenol red stands out, together with previous washes with PBS, followed by collection of the conditioned medium every 48–72 h, with storage at −80 °C [38]. Standardization of the methods of obtaining and processing secretome is essential to guarantee its reproducibility and efficacy in therapeutic applications, such as skin regeneration and immune modulation [28,48].

### 3.3. Methods for Secretome Production

The secretome of mesenchymal stem cells is commonly obtained from the conditioned medium of adherent cell cultures (2D or 3D). The culture media may contain fetal bovine serum (FBS), which is removed in the last stage of culture via a traditional method known as “starvation” [27,38]. The main challenge is maintaining homeostasis during prolonged culture at high cell density. Thus, in recent years, chemically defined media, such as MSC-XF from Rooster Nourish [49] and others, have appeared on the market and have also shown good performance both for expansion and obtaining high-quality conditioned medium [50]. Both 2D and 3D cultures show adequate secretion profiles of components participating in wound healing [31]; however, due to their spatial conformation, 3D cultures favor a greater production of secretome components than 2D culture systems [38,51]. Three-dimensional culture leads to improved cell interaction in the culture compared to monolayer cultures [31]. Hypoxic conditions of starvation cultures have also been reported to enhance secretome properties for wound healing processes, although some of them are reported under low FBS concentration conditions [38,41]. One strategy to improve the secretion profile is to use inducers, such as proinflammatory cytokines like IFN-γ and TNF-α, which suggest a beneficial paracrine effect on the secretion profile of MSCs for wound healing [33]. Under hypoxic conditions, this increases the production of angiogenic factors VEGF [44] and HIF-1α mediated by TGF-β1 [41]. The unconventional approach of using genetic modification through the transfection of AD-MSCs to overexpress angiogenic factors is noteworthy. This method has been tested and shown to enhance epithelialization, promote granulation tissue formation, and increase the expression of CD31, which indicates improved vascularization [25]. Additionally, it is important to note that culture conditions significantly influence various biological properties of MSCs, including their proteomes and immunomodulatory functions [2]. Furthermore, MSCs derived from different tissues display distinct epigenetic signatures [3].

#### 3.3.1. Influence of the Culture Media

Fetal bovine serum (FBS) is preferred in cell culture due to its richness in growth factors, essential nutrients, and key components that promote cell proliferation and development [52]. Additionally, FBS contains essential factors that support cell adhesion, an important aspect of maintaining the integrity and functionality of cultured cells [53]. However, from a regulatory standpoint, it is advisable to avoid using FBS for the production of cellular products or their derivatives intended for human use. The main concerns are the risk of zoonotic transmission and the variability in FBS batches, which can affect the quality of the cellular product [53]. Another alternative is the use of platelet lysate because it contains factors such as TGF-β, VEGF, and PDGF, which improve cell proliferation [39]. Nevertheless, using these supplements for secretome production may introduce exogenous materials, potentially compromising the quality and potency of the final product. On the other hand, the use of different xeno-free media can reduce variations in the composition of the cellular product, as well as its secretome, which can improve its quality and properties (Table 1). This allows for obtaining a more uniform and consistent secretome profile that is essential for therapeutic efficacy [1].

#### 3.3.2. Applications of Secretome in Wound Healing

Various strategies have been developed to enhance the therapeutic effectiveness of the secretome derived from mesenchymal stem cells (MSCs) in wound healing. A significant advancement in this area is the enrichment of the secretome with microRNA-146a [45]. Additionally, incorporating the secretome into hyaluronate sponges enables controlled release, further improving wound healing outcomes [22].

In vitro studies have provided valuable insights into the effects of secretome on cell lines [46]. Additionally, hydrogels infused with factors derived from human AD-MSCs have demonstrated significant benefits in wound healing, supporting the use of cell-free therapies [47]. On the other hand, in vivo studies have confirmed that the secretome from adipose tissue and umbilical cord MSCs promotes wound repair, angiogenesis, and dermal regeneration [25,27]. Furthermore, the development of spongy dressings that incorporate lyophilized MSC secretome has proven particularly effective in murine models, enhancing wound healing through various proteomic interactions [39].

Recent advances in the production of secretome, particularly through the optimization of maturation processes, have enhanced their effectiveness in promoting scarless healing [38]. Human mesenchymal stem cell (MSC)-conditioned medium also influences the behavior of fibroblasts, which is crucial for healing diabetic wounds [48]. Additionally, combining MSC secretome with umbilical cord platelet lysate has demonstrated synergistic effects in treating chronic wounds [28]. However, certain applications, such as skin-derived hydrogels containing adipose-derived MSC (AD-MSC) secretome, have shown limited effectiveness in skin flap regeneration [55] (Table 2). Hydrogel-based strategies have been extensively investigated. For instance, extracellular matrix/alginate hydrogel patches that are enriched with MGTFVC SC secretomes can accelerate the wound healing process [42]. Additionally, pretreating MSCs with pro-inflammatory cytokines enhances their ability to facilitate wound healing by promoting macrophage migration and VEGFC-mediated angiogenesis [33,34] (Table 2). Comparisons between secretomes derived from umbilical cord MSCs and dental pulp MSCs have offered valuable insights into their metabolomic profiles and functional mechanisms [24].

In addition to promoting wound healing, secretome derived from MSCs has demonstrated protective effects against venom-induced dermonecrosis and plays a crucial role in blood vessel formation and vascular stability during skin repair [25,28]. The TNF-α-induced secretome has also unveiled key biological processes that are essential for effective wound healing [7]. Recent advances have led to the development of new hydrogels, such as those made from fusion proteins (including spider silk and squid suckerin), which incorporate RGD (arginine–glycine–aspartic acid) peptides and release the secretome in a controlled manner, ensuring therapeutic levels are maintained over time [56]. Another promising approach involves using collagen hydrogels as a scaffold for the supernatant from hAD-MSC cells. This combination has shown proangiogenic and antimicrobial effects, thereby enhancing angiogenesis and inhibiting bacterial growth [47]. These hydrogels serve as effective carriers for the secretome, preserving its biological activity while exhibiting regenerative, immunomodulatory, and antimicrobial properties [29,45,47].

The secretome of MSCs has been extensively studied for its effects on cellular mechanisms involved in wound healing, and synthetic extracellular matrices have emerged as effective tools to enhance the therapeutic effects of the MSC secretome [57,58]. Additionally, the combination of low-level pulsed wave laser therapy with MSC bone marrow-conditioned medium has demonstrated synergistic effects in healing diabetic wounds [43]. Three-dimensional MSC spheroid cultures have also been shown to enhance paracrine signaling [10]. The use of tissue-mimetic culture systems further improves the capacity of the MSC secretome, promoting the regeneration of keratinocytes and fibroblasts [49]. Hyaluronic acid-based hydrogels that incorporate MSC secretome have successfully facilitated the repair of diabetic ulcers [35]. Furthermore, comparative studies have highlighted the healing potential of MSC-conditioned media derived from adipose tissue [59]. Overall, the MSC secretome demonstrates significant promise in enhancing wound healing processes through various innovative approaches.

**Table 2:** Current state of secretome use in practical applications (in vivo).

Source Secretome	MSC Preconditioning	In Vivo Application	Method of Analysis			Delivery	Key Outcomes	Ref.
			Gross (Macroscopic)	Histology	Molecular Secretion			
hBM-MSC	Hypoxia	Diabetic wound	Gross morphology	HE, immunofluorescence for markers such as K14 and ATG5/7 Histomorphometry	Autophagy (ATG5, LC3B) and signaling factors (TGF-β1, SMAD-2)	Intradermal injection	Hypoxic hBM-MSC increased epidermal cell autophagy, proliferation, and migration through TGF-β1. HIF-1α/TGF-β1/SMAD axis signaling significantly enhanced re-epithelialization and wound healing in diabetic models. TGF-β1 interference reduced these effects, highlighting its central role.	[41]
hAD-MSC	None	Skin ulcer	Gross morphology	HE, MT	BioPlex: Angiopoietin-2, G-CSF, HGF, PDGF-BB, and VEGF	Injection	Cell sheets accelerated ulcer closure and promoted dermal regeneration with the recovery of skin appendages, such as hair follicles and glands. The secretome showed positive effects on the vascularization of granulation tissue, although less effective than cell sheets in inducing complete regeneration.	[26]
hUC-MSC	Serum-free medium	Psoriasiform dermatitis	Gross morphology and morphometry, PASI	HE	IL-17A, TNF-α	Topical in hyaluronic acid scaffold	Treatment with secretome-loaded HA sponges significantly reduced IL-17A and TNF-α levels.	[46]
hUC-MSC	None	Diabetic and trophic ulcer	Gross morphology and morphometry	ND	ND	Topical in 10% SM-hUC-MSC gel	Significant reduction in ulcer size (length, width, and area) after treatment with secretome gel.	[29]
hAD-MSC	VEGF-A and Hif-1α transfection	Cutaneous wound	Gross morphology	HE	ND	Injection: sub-dermal dose	Enhanced epithelialization and granulation tissue formation in groups treated with the enriched secretome. Higher expression of CD31, improved vascularization.	[60]
hUC-MSC	Xeno-free medium culture	Full-thickness excision wound	Gross morphology	HE	ELISA: IL-6, IL-10, IFN-γ, and TNF-α	Injection: subdermal	Higher wound closure rate and lower inflammation in the groups treated with hUC-MSC and CM. Higher collagen density, better organization in scar tissue.	[27]
hADMSC	None	Cutaneous wound	Gross morphology	HE, tMA	LC-MS/MS: decorin, tenascin, glyceraldehyde-3-phosphate dehydrogenase 1 glyceralde, fibrinogen, factor XIII, annexin A1, collagen type I	Topical in collagen hydrogel lyosecretome	Secretome-containing dressings promote faster and more complete regeneration, increased re-epithelialization, vascularization, granulation tissue, and collagen deposition. Key proteins such as Decorin, Tenascin, and the epidermal growth factor receptor (EGFR) are overexpressed in treated wounds, suggesting their role in skin repair and remodeling.	[39]
hADMSC	None	Skin wound (Puncture wound)	Gross morphology	HE, TUNEL staining	VEGF	Intraperitoneal injection and transdermal (iontoferesis and metal roller)	The secretome promotes healing without toxic response, enhances angiogenesis, and can be effectively applied transdermally.	[38,39]
hBM-MSC	None	Diabetic wound	Gross morphology	HE, tMA	EGF and bFGF	Lyosecretome	Conditions and migration of fibroblast-enhanced wound closure. Improves vascularization and remodeling of granulation tissue in diabetic wounds.	[48]
hAD-MSC	None	Skin wound (Wound perfusion)	Gross morphology	HE	ND	Topic in EMC-secretome	Improves angiogenesis in ischemic areas, but without significant improvement in healing.	[55]
hBM-MSC	None	Full-thickness skin wound	Gross morphology	HE, Herovici Staining	ND	Topical (EMC alginate hydrogel patch secretome)	Secretome with hydrogel patch significantly accelerates healing.	[42]
hUC-MSC	IFN-γ and TNF-α (IT)	Skin excision wound	Gross morphology	HE, IF	ELISA: IL6 and CCL2	Subcutaneous injection	S-IT MSCs significantly enhanced macrophage migration and polarization towards the M2 phenotype. This led to improved wound closure and overall healing efficiency, underscoring the therapeutic potential of the S-IT MSCs secretome in promoting skin regeneration	[33]
hUC-MSC	IFN-γ and TNF-α (IT)	Skin excision wound	Gross morphology	HE and immunohistochemistry	ELISA: VEGFC	Topical	The S-MSCs-IT significantly accelerated wound closure compared to control groups (S-MSCs, control medium).	[34]
hAD-MSC	None	Dermonecrosis	Gross morphology	HE	ND	Intradermal Injection and intravenous route	The MSC secretome demonstrated protective effects against dermonecrosis induced by Loxosceles intermedia spider venom. Animals treated with the secretome showed signs of tissue healing, such as fibroblast activation, neovascularization, and tissue re-epithelialization, especially in the group that received intravenous administration.	[40]
hAD-MSC	Hypoxic	Full-thickness skin wound	Gross morphology	ND	ND	Subcutaneous injection	The study found that hAD-MSC secretomes significantly accelerated wound healing, increased endothelial density, and enhanced pericyte coverage (specifically expressing NG2 and nestin).	[37]
hAD-MSC	hTERT immortalized	Diabetic wound	Gross morphology	HE and picrosirius red (PSR)	ND	Topical in hydrogel NSC-2R-secretome	Accelerated wound closure rates for groups treated with the secretome-laden hydrogel compared to control groups. Increased endothelial cell proliferation, which aids in angiogenesis and tissue regeneration. A reduced pro-inflammatory cell population in treated wounds indicates effective immunomodulation and improved healing dynamics.	[56]
hAD-MSC	None	Excisional cutaneous wound	ND	ND	KI67 and CD31	Topical	The stem cell secretome shows accelerated wound healing, characterized by increased skin cell proliferation and migration, increased dermal and epidermal thickness, enhanced angiogenesis (increased CD31 expression), and an overall reduction in scar formation.	[58]
hBM.MSC	None	Diabetes mellitus	Gross morphology	ND	ND	Injection	Significant improvement in wound closure rates compared to control groups. Enhanced biomechanical properties of the healed tissue. Increased presence of fibroblasts and blood vessels in the treated wounds, indicating improved tissue repair and regeneration.	[43]
hUC-MSC	3D culture	Skin injury	Gross morphology	HE	EGF-A, TGF-β1, KGF, FGF-2, IL-6, G-CSF, and HGF	Topical	Improved fibroblast-mediated extracellular matrix (ECM) synthesis. Promotion of angiogenesis and vasculogenesis, essential for granulation tissue formation and remodeling during wound healing.	[31]
hBM-MSC	None	Diabetic wound healing	Gross morphology	ND	ND	Topical—lyosecretoma	A significant improvement in wound closure rates was observed in the groups treated with hBM-MSC secretome and PWLLLT compared to the control group. In the groups receiving combined treatment (hBM-MSC-CM + PWLLLT), wound closure was more pronounced than in the groups receiving only one of the treatments.	[61]
hUC-MSC	None	Skin ulcer	Gross morphology	HE, immunofluorescence and image analysis	ND	Topical (hyaluronic acid (HA)–secretome)	Significant reductions in the wound area and improved rates of re-epithelialization in ulcers treated with the hydrogel loaded with either hUC-MSC secretome compared to the control (hydrogel only or vehicle).	[35]
hAD-MSC	None	Skin wound	ND	ND	Collagen type I and metalloproteinase-1	Topical	Higher rates of wound healing, as evidenced by smaller wound areas in ATSC-Ex-treated wounds compared to controls on days 4, 6, and 8, and near-complete closure on day 12.	[62]

Human umbilical cord-derived mesenchymal stem cells, h**UC-MSC**; **human** adipose tissue-derived mesenchymal stem cells, h**AD-MSC**; human bone marrow-derived mesenchymal stem cells, h**BM-MSC**; hematoxylin and eosin, **HE**; Masson’s trichrome, **MT**; not done, **ND**; psoriasis area severity index, **PASI;** Azan Mallory trichrome, **tMA**; immunofluorescence, **IF**; low-level pulsed wave laser therapy, **PWLLLT**; human bone marrow mesenchymal stem cell-conditioned media, h**BM-MSC-CM**; keratin 14, **K14**; autophagy protein, **ATG5/7;** autophagy protein, **LC3B**; transforming growth factor beta 1, **TGF-β1**; signaling pathway, **SMAD-2**; hypoxia-induced factor-1 alpha, **HIF-1α**; granulocyte-colony stimulating factor, **G-CSF**; hepatocyte growth factor, **HGF**; platelet-derived growth factor, **PDGF-BB**; vascular endothelial growth factor, **VEGF**; vascular endothelial growth factor c, **VEGFC**; interleukin-17A, **IL-17A**; tumor necrosis factor alpha, **TNF-α**; tumor necrosis factor beta, **TNF-γ;** hyaluronic acid, **HA**; secretome of human umbilical cord mesenchymal stem cells, **SM-hUC-MSC**; platelet endothelial cell adhesion molecule, **CD31**; interleukin-6, **IL-6**; interleukin-10, **IL-10**, **IFN-γv**; conditioned media, **CM**; liquid chromatography–tandem mass spectrometry, **LC-MS/MS**; epidermal growth factor receptor, **EGFR**; extracellular matrix, **EMC**; initiation and termination, **IT**; secretome mesenchymal stem cells initiation and termination, **S-MSCs-IT**; secretome initiation and termination, **S-IT**; neuron glia antigen-2, **NG2**; immortalized cell, **hTERT**; arginine–glycine–aspartate-presenting triple-species chimeric fusion protein, **NSC-2R**; three-dimensional, **3D**; keratinocyte growth factor, **KGF**; fibroblast growth factor 2, **FGF-2**.

- **Clinical Trials with Secretome** 

Research on the effect of secretome in wound healing is a relatively new field, but significant advancements have been made thanks to technological progress. Recent studies from 2023 have reached the clinical phase [29,61]. In the first study, a topical gel containing 10% hUC-MSC secretome was applied twice a day for two weeks to patients with leprosy and diabetes mellitus. The study has been registered at ClinicalTrials.gov with ID number: NCT04134676. The treatment resulted in a significant reduction in wound dimensions: the length was reduced by 37.5%, width by 38.5%, and area of chronic wounds by 54.8% [29]. A second study utilized conditioned medium from adipose-derived mesenchymal stem cells in patients with leprosy, with Trial Registration Number: 0052/LOE/302.4.2/VII/2020. After eight weeks of topical treatment applied every three days, the researchers observed an impressive reduction in both the size and depth of wounds by 82% and 95.8%, respectively. In some cases, complete wound closure was achieved. The study also demonstrated that the efficacy of the adipose MSC-conditioned medium was superior to traditional framycetin gauze dressings [61]. These findings are promising for the development of secretome-based products, and, importantly, no adverse reactions were reported with this therapy, indicating its safety for clinical applications.

- **Current State of Secretome Use in Commercial Practice** 

Research into the secretome of mesenchymal stem cells (MSCs) has made significant strides, leading companies involved in research and development (R&D) innovation to create novel and highly effective products for the public. Currently, various products on the market utilize secretome to address a range of conditions, including skin care, alopecia, anti-aging, psoriasis, and wound healing, as detailed in Table 3 [63,64,65,66]. However, dressings that incorporate secretome for treating chronic ulcers are still in the research and development phase. Notably, the Viennese company APOSCIENCE has developed a gel called APOSEC for treating poorly healing diabetic foot wounds; this gel is based on secretome derived from peripheral blood mononuclear cells and is manufactured following good manufacturing practices (GMP). The study has been registered at ClinicalTrials.gov with ID number: NCT04277598 [67]

## 4. Discussion

The secretome derived from mesenchymal stem cells (MSCs) is emerging as a valuable tool in regenerative medicine [14]. As research advances, it becomes increasingly evident that the immunomodulatory and regenerative properties of secretome result from a complex combination of bioactive factors, including cytokines, exosomes, and soluble proteins [14,15,17]. However, the effects of the secretome are not consistent; studies indicate that its efficacy varies depending on the cellular source it comes from and the clinical context in which it is used. These variations raise important questions and challenge the scientific community to further investigate the mechanisms that define its true therapeutic potential [16,18].

When examining various sources of mesenchymal stem cells, it is clear that those derived from adipose tissue (AD-MSC) and umbilical cord (UC-MSC) have attracted the most research interest. In contrast, bone marrow-derived MSCs have received less attention in this specific context. This difference may be due to the practical and ethical advantages offered by UC-MSCs and AD-MSCs, which include non-invasive collection methods and a high capacity for proliferation [17]. Furthermore, the secretome of UC-MSCs has been associated with enhanced angiogenic effects, attributed to its high levels of VEGF and HGF. These factors are particularly advantageous for chronic wound healing [16,18,19,21,22,25].

Optimization of the secretome of mesenchymal stem cells (MSCs) has been an area of growing interest due to its therapeutic potential in tissue regeneration. The choice of cell source significantly influences secretome composition and functionality. BM-MSCs are cultured in DMEM with 0.1% FBS, under normoxic or hypoxic conditions, impacting the release of angiogenic factors [26,41]. In contrast, AD-MSCs require longer incubation times (48 h to 7 days) and additional processing, such as filtration, ultrafiltration (3–5 kDa), and exosome isolation, to maximize their effectiveness [30,36,45]. On the other hand, UC-MSCs, cultured in DMEM without phenol red and stored at −80 °C, are a promising source due to their high secretome yield and immunomodulatory profile [29,46]. To improve secretome stability and functionality, various processing methods have been implemented. Centrifugation (1500–3000× *g*) is used to remove cellular debris, while ultrafiltration and lyophilization are effective strategies to concentrate bioactive factors and facilitate their storage [25,27,39]. Furthermore, hypoxia (5% O_2_) in BM-MSCs and UC-MSCs has been shown to enhance the secretion of VEGF and bFGF, promoting angiogenesis and tissue repair [38,47]. In the case of AD-MSCs, enrichment of the secretome with exosomes using techniques such as ExoQuick-TC and NanoSight LM10 allows the evaluation and enhancement of their content in extracellular vesicles, essential for cellular communication and regeneration [28,48]. Beyond trad itional methods, advanced approaches have improved secretome functionality. Genetic manipulation of MSCs has allowed the overexpression of key factors, such as VEGF-A and HIF-1α, to be induced, optimizing their regenerative capacity [48]. Furthermore, the use of biomimetic hydrogels for controlled secretome release has shown efficacy in chronic wound models [44], while the combination with three-dimensional matrices and collagen scaffolds has increased their stability and biological activity [33,42].

In clinical terms, the development of ready-to-use formulations has been key in the translation of secretome to regenerative medicine. Recent studies have shown that secretome-enriched gels (10% SM-hUCMSC gel) are effective in the treatment of chronic ulcers and diabetic wounds, highlighting the importance of their standardization to ensure reproducibility and efficacy [24,32,34,37,40,56]. Furthermore, the combination of secretome with biomaterials such as hyaluronic acid hydrogels has shown promising results in regenerative therapies [57,58].

Secretome collection and processing have evolved towards more sophisticated methodologies that allow for improved stability, bioavailability, and clinical application. However, the variability in protocols underlines the need for standardization, which would ensure their reproducibility and efficacy in medical applications [29,34,43,49,59,61,62,68,69].

Understanding how secretome operates is crucial for unlocking its therapeutic potential [41]. Among the various molecular pathways involved, the HIF-1α/TGF-β1/SMAD and NF-κB pathways are particularly significant due to their roles in inflammation and tissue regeneration. These pathways offer new opportunities for enhancing clinical applications [41,52]. However, a key challenge is the variability in the composition of the secretome, which can affect its therapeutic effectiveness. To tackle this issue, researchers have employed immortalized cell lines, such as HATMSC [47] and CRC-4000 [36], to produce a more consistent secretome. While this approach helps standardize preclinical trials, it raises the question of whether these models can accurately mimic the effects of the secretome in actual clinical settings [11].

Culture media are crucial for obtaining the secretome derived from mesenchymal stem cells (MSCs), as they directly influence its composition and properties [27,31,37]. Chemically defined media offer a way to standardize MSC-derived products and their applications in regenerative medicine. These media create a controlled environment that encourages the secretion of specific proteins in the MSC secretome, while also eliminating the risk of contamination from animal-derived factors. This is vital for ensuring safety and reproducibility in cell therapy [50,52]. Consequently, using chemically defined media guarantees greater consistency in product quality and reduces variability between different batches, which is essential for the therapeutic use of the secretome.

An important factor to consider is the use of specific components in culture media, as these can significantly influence the bioactive properties of the secretome. Adding supplements, such as growth factors and cytokines, can enhance the ability of mesenchymal stem cells (MSCs) to secrete proteins that possess immunomodulatory and regenerative activity [50]. This indicates that media formulations should be finely tuned based on the type of MSC and the intended therapeutic application. Conversely, the impact of additives in the medium also affects secretome production. Research has shown that certain factors, like glucose and specific amino acids, can promote the secretion of proteins related to wound healing, thereby creating new opportunities for clinical applications in regenerative medicine and tissue repair [32,40].

In the field of advanced manufacturing, research has explored the optimization of culture media for large-scale secretome production using bioreactor systems and three-dimensional technologies. Recent studies have shown that the integration of three-dimensional matrices and chemically defined media, such as RoosterNourish XF and beadMATRIX, leads to an increase in the secretion of proteins with improved properties, thereby optimizing secretome production at an industrial scale [68]. This research not only increases the quantity of secretome produced but also enhances its quality and functionality. Advances in biotechnology have also paved the way for the development of culture technologies that support the production of mesenchymal stem cells (MSCs) under fully defined conditions [52,53]. The XFS patent, for instance, describes a culture medium specifically designed for MSCs that not only promotes cell proliferation but also enhances the efficiency of bioactive factor secretion. This breakthrough is crucial for large-scale secretome production, making regenerative therapies more accessible and effective [42].

Three-dimensional (3D) culture optimizes the adhesion, proliferation, and secretion of mesenchymal stem cell (MSC) secretome by recreating a biomimetic cellular microenvironment that activates key signaling pathways [70,71,72]. Among the involved mechanisms, the FAK/Src pathway regulates cell adhesion and mechanotransduction, promoting MSC survival and proliferation [71]. Likewise, MAPK drives cell proliferation and differentiation, while PI3K/Akt inhibits apoptosis and stimulates the production of growth factors and cytokines, enhancing the secretome [71,73,74]. Additionally, Wnt/β-catenin contributes to self-renewal and pluripotency, whereas YAP/TAZ reinforces mechanotransduction and the expression of bioactive genes [71,74,75,76]. The use of electrospun scaffolds in 3D culture enhances cell adhesion through polymers that increase hydrophilicity and facilitate integrin activation [71,77], triggering signaling cascades that optimize MSC functionality [77]. These scaffolds also promote the secretion of key bioactive factors, such as bFGF, HGF, iNOS, PGE2, TGF-β, and VEGF, which are fundamental for their therapeutic potential [71,78].

Secretome has shown a remarkable ability to promote tissue repair, reduce inflammation, and enhance angiogenesis, making it an effective treatment for various skin injuries in both animal models and clinical applications in humans [25,26,27,31,33]. Studies using animal models have demonstrated accelerated healing of chronic wounds and improved tissue regeneration. The efficacy of the adipose-derived mesenchymal stem cell (AD-MSC) secretome in particular has been highlighted in the treatment of diabetic ulcers and pressure sores, where it promotes angiogenesis and the formation of granulation tissue [25,26].

The therapeutic effect of stem cells is enhanced when they are cultured in three-dimensional conditions, which increases the release of bioactive factors that are crucial for regeneration [28]. Moreover, preconditioning mesenchymal stem cells (MSCs) with pro-inflammatory cytokines has been shown to accelerate healing by facilitating the migration and polarization of macrophages towards an anti-inflammatory (M2) phenotype. This shift is essential for preventing excessive scarring [31]. Additionally, the topical application of secretome has yielded promising results; hydrogels infused with secretome have been used in the treatment of diabetic ulcers, demonstrating accelerated healing and improved regeneration of the affected tissues [33]. These findings underscore the significant potential of the secretome in enhancing the repair of various types of wounds, from chronic to complex, thereby greatly contributing to tissue healing.

In clinical settings, the effectiveness of mesenchymal stem cell (MSC) secretome in wound healing has recently been evaluated, highlighting its therapeutic potential for various conditions. The primary mechanism of action involves the release of bioactive factors that modulate inflammation, regulate immune responses, and stimulate cell proliferation, ultimately promoting tissue repair [29]. Additionally, a comparative clinical trial assessed the efficacy of MSC secretome against gauze dressings impregnated with framycetin for treating chronic leprosy plantar ulcers. The results showed that patients treated with secretome experienced faster healing and improved re-epithelialization [61]. These findings underscore the role of MSC secretome as an innovative therapeutic strategy in regenerative medicine, with the potential to revolutionize the treatment of chronic wounds and pathologies related to tissue dysfunction. This approach offers an effective and less invasive alternative to conventional therapies [29].

The commercial application of secretome-based products is gaining traction, especially in skincare, due to its regenerative and anti-inflammatory properties. Products such as Adiposecr ^TM^ utilize AD-MSC secretome to improve skin hydration, elasticity, and regeneration [65]. Similarly, Carmell Secretome ^TM^ employs secretome-derived components to combat skin aging by promoting repair through growth factors [64]. Meanwhile, CF-FECS-DF by SBiomedics stands out for its anti-inflammatory and regenerative effects, enhancing skin elasticity and reducing wrinkles [63], and Exostem4Tech ^®^ uxostem4Tech ^®^ improves hydration and reduces signs of aging based on exosome-derived secretome to strengthen the skin barrier [63]. These products demonstrate secretome’s potential, not only in skincare, but also in future applications for regenerative medicine about cutaneous wound healing.

## 5. Conclusions

Secretome derived from mesenchymal stem cells (MSCs) is increasingly recognized as a promising tool in regenerative medicine, particularly for the treatment of chronic wounds and tissue repair. Its immunomodulatory and regenerative properties, which are mediated by bioactive factors, such as cytokines, exosomes, and soluble proteins, offer innovative solutions for various complex pathologies, from skin injuries to tissue dysfunction disorders. However, there are challenges to overcome, such as the variability in secretome composition and clinical outcomes, which underscore the need for a deeper understanding of the underlying molecular mechanisms. Differences between MSC sources—such as adipose tissue and umbilical cord—along with advancements in culture media are critical factors that can enhance the consistency and efficacy of secretome in clinical applications.

More research into advanced culture systems and the optimization of production in bioreactors are essential steps toward standardizing and expanding the therapeutic use of secretome. With ongoing validation through preclinical and clinical trials, secretome has the potential to revolutionize wound treatment and play a significant role in regenerative medicine, both in clinical practice and cosmetic applications.

## Figures and Tables

**Table 1:** Xeno-free chemically defined media used for obtaining secretome.

Medium	Content	Tested on	Ref.
UrSuppe	Defined molecules, recombinant human growth factors, injectable albumin	hAD-MSC	[53]
Oxium	Defined molecules and antioxidants	hUC-MSC for extracellular vesicle production	[50]
Xanadu	Defined molecules	hAD-MSC for secretome production against Loxosceles intermedia venom	[40]
MSC-XF from Rooster Nourish	Defined molecules	hUC-MSC and hBM-MSC	[49]
XFS (patent No. PCT/EP2015/053223)	MEM-u, ascorbic acid 2-phosphate, dexamethasone, lipoprotein, human serum albumin (HSA), ITS	MSC	[54]

Human adipose tissue-derived mesenchymal stem cells, h**AD-MSC**; human bone marrow mesenchymal stem cells, **hBM-MSC;** human serum albumin, **HSA**; insulin–transferrin–selenium, **ITS**; human umbilical cord-derived mesenchymal stem cells, h**UC-MSC**; mesenchymal stem cells, **MSCs**; minimum essential medium, **MEM**.

**Table 3:** Secretome products available on the market for skin conditions.

Manufacturer	Location	Product	Source	Application	Ref.
Secretosome	Tehran, Iran	Adiposecr ^TM^	Secretome from hAD-MSC	Hyperpigmentation; dull, tired skin; wound healing; post-inflammatory hyperpigmentation; sun damage; rosacea and redness; dehydrated skin; chemical peel aftercare	[65]
Carmell	Pittsburgh, Pennsylvania, USA	Carmell Secretome ^TM^	Secretome from hBM-MSC	Anti-aging, dark spots, redness	[64]
S.Biomedics	Seoul, Seongdong-gu, Republic of Korea	CF-FECS-DF	Secretome from human dermal fibroblasts spheroids	Damaged skin	[63]
Falonlabs	Granada, Spain	Exostem4Tech ^®^	Secretome from MSC.	Skin wound	[66]

Human adipose-derived mesenchymal stem cells, h**AD-MSC**; human bone marrow-derived mesenchymal stem cells, **hBM-MSC.**
